# Observations on the Employment of Chloruret of Oxide of Calcium in the Treatment of Purulent Ophthalmia

**Published:** 1827-11

**Authors:** 

**Affiliations:** Surgeon to the Military Hospital, Brussels, &c. &c.


### PURULENT OPHTHALMIA.

*2, Berkeley-street; August* 20, 1827.

DEAR SIR,—I send you herewith a letter addressed to me by Dr. VARLEZ, surgeon-major of the Military Hospital at Brussels, on the use of the Chloruret of the Oxide of Calcium, which he seems disposed to consider nearly as a specific in cases of Purulent Ophthalmia. I have added three cases, selected from many others, which prove its utility, although at the same time demonstrating the necessity for its being accompanied by severe depletion in every instance in which the eyeball is affected. It may perhaps be useful for some of your readers to know that the purulent ophthalmia of *infants* is a much less serious disease than that of adults, and that there are two kinds of it : one affecting the lids, the other implicating the eyeball. The danger, both in infants and in adults, is of course in proportion to the affection of the cornea, although in infants it is less than in children or in adults, where the extent of disease is apparently the same.

I am, dear sir, your obedient servant,

G. J. GUTHRIE.

Dr. MACLEOD.

*Observations on the Employment of Chloruret of Oxide of Calcium in the Treatment of Purulent Ophthalmia.* By Dr. VARLEZ, Surgeon to the MILITARY HOSPITAL, BRUSSELS, &c. &c.

YOU have, without doubt, as well as myself, lamented the unfortunate condition of those affected with purulent ophthalmia in a severe form, and you will therefore learn with pleasure the favourable results which I have obtained in more than 400 patients, by means of a remedy which up to this time has not once disappointed me. Aware that you have paid attention to this fatal disease, I send you an account of my experience with the chloruret of oxide of calcium, in hopes that you will give it a trial, and make the profession acquainted with the results.

I have treated, during more than ten years, a prodigious number of patients affected with ophthalmia in the military hospitals of the Netherlands, and have seen the disease in all its forms, beginning mildly or with various alarming symptoms, and I have often had occasion to be convinced that it is extremely dangerous when accompanied with purulent secretion, sometimes producing ulceration and destruction of vision, in spite of the most vigorous and rational treatment. I have seen the suppuration continue, and the inflammation produce frightful disorganisation, in soldiers who had been bled ten or twelve times in the arm, temporal artery, or jugular vein. I have seen the most active purgatives, calomel in considerable doses, blisters, sinapisms, and setons, various ointments, together with every variety of collyrium, all fail. I have more than once treated the disease with all these various remedies, and the blindness of the patient which has supervened has attested the energy of this destructive malady, and the impotence of art. In this state of matters, appointed by the good opinion of the principal medical officer of our army to the hospital in Brussels, where the ophthalmic patients are very numerous, and where, just before my arrival, two men had lost their sight, I sought some means calculated to change the mode of vitality of the diseased part, and to suppress the formation of matter, the contact of which on the cornea produces softening, ulceration, and even perforation, sometimes in the course of a few hours. Having been struck with the rapidity with which the chloruret of oxide of calcium, when applied to the ulcers, diminished the suppuration, I directed all my attention to this valuable agent, and it has answered so well to my expectations, that I think I may safely declare that it is the best remedy known, not only in the purulent ophthalmia of adults, but in blennorrhœa and the purulent ophthalmia of children, as well as in all those cases which, having passed into the chronic state, are accompanied with mucous secretion, or disease of the meibomian glands.

In order to enable you to judge of our ophthalmia, and of the success which I have witnessed from the solution of the chloruret, I shall relate some cases, which, if I mistake not, will convince you that the results which I have obtained merit some attention.

Case I.—Ciswiski, a musician, thirty-four years of age, of the lymphatico-sanguine temperament, admitted into the Military Hospital of Brussels, in November 1826, with the following symptoms:—The eyelids red, much swollen, and closed; the conjunctiva of the eye and eyelid tumefied, the first forming a projection round the cornea, which was quite hid; a thick and glutinous suppuration flowed over the cheeks; the patient was tormented with intense headache over the brow; the skin was hot, the pulse hard and frequent, although he had been bled before his arrival at the hospital, by the medical officer at his barrack, who was afraid of mischief speedily supervening. I bled him from the temporal artery to the extent of nineteen ounces. I gently raised the upper eyelid, and depressed the lower, in order to apply to the diseased conjunctiva the solution of the chloruret, which I introduced with a hair pencil, because I could not separate the edges of the eyelids sufficiently to make the application in any other way. This was repeated every three hours, and immediately after the eye was covered with a compress soaked in very cold rain-water, frequently renewed to keep up the coolness.

In six hours, the suppuration had diminished: it was distinctly less glutinous, and not so thick. I took ten ounces of blood from the arm; continued the solution of the chloruret and cold compress as before.

Next day, the suppuration was almost suppressed; the tumefaction considerably diminished, and the patient able to open his eyes. The solution continued, being dropt into the eye eight times in the twenty-four hours.

Twelve days after his admission, the patient left the hospital perfectly cured.

In this case I employed half a drachm of the chloruret to an ounce of distilled water, a fresh solution being made every day.

Case II.—Preisser, a soldier, was admitted into the Military Hospital, the 19th of November, 1826, having been violently stimulated by a prodigious quantity of wine, and having passed the night out of the barrack. He states that his eyes were heated, and became inflamed on the morning of the 19th. On his admission, I found him with the eyelids very much swelled, as if injected with serous fluid; the margin smeared with a tenacious and adhesive suppuration; the eyes shut, and unable to bear the light even through the lids; the conjunctiva of a scarlet colour; the pain severe, extending to the forehead, and accompanied with violent beating of the temporal arteries.

**Bled to twelve ounces from the temporal artery. Solution of the Chloruret to be dropped into the eye three times a-day. Cold lotion.**

16th.—The swelling of the eyelids is diminished; the pain completely gone.

**The solution to be used five times a-day.**

Next day, he was still better; and on the 19th he was discharged.

In this case the chloruret was employed in the proportion of a scruple to an ounce of distilled water; but when the inflammation continues, and when the patients bear the application without complaining, I push the quantity to three or even four drachms in the same quantity of water; and where the eyelids are very much swollen, so that I cannot introduce the solution in such a manner as to cover all the surface of the conjunctiva, I inject it with a small syringe.

Case III.—The infant of M. Crouse, a merchant in this town, was attacked with a severe purulent ophthalmia three weeks ago. I applied the solution of the chloruret, in the proportion of ten drops to the ounce of water, four times a-day. In three days he was quite well.

Case IV.—The infant of M. Forten, bookseller, had laboured for more than a year under irritation of the eyes, accompanied with considerable secretion from the meibomian glands. She had been under treatment at Paris for a long time, without any benefit, and yet she was cured in eight days. The solution was used three times a-day.

I have treated with the same remedy a considerable number of cases of purulent ophthalmia, accompanied by granulations, mucous secretion, and an injected condition of the conjunctiva and cornea, and up to this time the remedy has always succeeded. I do not, however, suppose that it can enable the practitioner to dispense with the other resources of art, when they are indicated by the state of the disease: it is thus that in severe acute ophthalmia I employ copious general bleeding, frequently repeated, purgatives, calomel, pediluvia, &c.; while in chronic cases I use setons, blisters, &c. conjointly with the solution of the chloruret.

I intend in a short time to publish a work on the ophthalmiæ of our army, and to enter into all the details which relate to the treatment of this formidable disease. At present I shall content myself with adding, that when I perceive that the solution of the chloruret ceases to be of service in chronic ophthalmia, I either augment the quantity or suspend its use, to be again resumed after a time.

The following are three cases in which the remedy was tried by Mr. Guthrie, at the Royal Westminster Infirmary for Diseases of the Eye:

Case I.—Peter Savage, a bricklayer's apprentice, aged twenty, applied at the Infirmary, May 28th, with purulent ophthalmia, which came on two days before, without any cause that he could assign, or that could be ascertained. The discharge was considerable; the eyelids red, swelled, and closed. On elevating the upper eyelid, the cornea appeared sound, but surrounded and encroached upon by the conjunctiva, which was raised around it in a state of chemosis; the pain in the eye, forehead, and side of the head, great.

V.S. ad ℥xxxvj.—Pil. Cal. gr. vj.; Magnes. Sulph. ℥j.—The eye to be kept constantly wet and cold.

On the 29th I first saw him, and directed the bleeding to be repeated to thirty ounces; and the solution of the chloruret of the oxide of calcium, as sold at Garden's, to be applied three times a-day with a brush to the conjunctiva; the cold water and the purgatives to be continued.

30th.—The external remedies and the purgatives to be repeated, and a blister applied behind the ear.

31st.—The chemosis has greatly subsided; he has no pain; the discharge from the eye is less, the whole of the cornea cannot yet be seen; the swelling of the lid continues.

To be cupped to sixteen ounces.

June 1st.—There being no improvement, but rather a slight increase of pain, fourteen ounces of blood were taken from the arm.

2d.—Better; but, as the pain is not entirely gone, to be bled again to fourteen ounces.

4th.—A return of the pain having taken place, from using the other eye, and attempting to do some work, he was bled to sixteen ounces.

12th.—Continued to improve until this day, when, from having attempted to work, he brought on a recurrence of pain.

To be cupped to fourteen ounces.

21st.—The last remark is—this case is an excellent one, and the cure perfect, there being no defect whatsoever.

The only remedies used were the abstraction of blood, constant purgatives, cold water, and the solution of the chloruret of the oxide of calcium, with a little common ointment applied to the edges of the lids at night.

Case II.—Ann Leacock, aged two years, of emaciated and unhealthy appearance, and having her head covered with tinea, was brought to the Infirmary on the 17th July, labouring under purulent ophthalmia of the right eye, of four days' duration. The lids were firmly agglutinated, and, when separated, a stream of yellow greenish-coloured fluid was discharged. There was much chemosis, but, from the violence of the child's efforts, it was impossible to examine accurately the state of the cornea. She had suffered from measles four weeks previous to her admission at the institution; since her convalescence from which, her eyes had been in a weak state.

To be cupped to ℥iv. from the temple.—℞. Submur. Hyd. gr. iij. o. nocte.—Infus. Sennæ ℥jss. cum Sulph. Mag. ℥j. mane.—Lids to be everted, and the Sol. Oxyd. Chl. Calc., diluted with equal parts of water, to be applied morning and evening. In the intervals, the eye to be frequently syringed with the Lotio Aluminis, and to be repeatedly fomented with warm water.

21st.—Inflammation so far subdued as to permit the accurate inspection of the cornea, which was found to be quite transparent; the conjunctiva very vascular, but not granular. Her bowels had been steadily acted upon, and the general health much improved.

Two leeches to lower lid. Blister behind the ear. Continue the pills and purgative draught.

25th.—Her gums have become tender, and her breath fetid.

Omit the pills. Continue the other remedies.

31st.—Still some discharge, though of a whitish and more healthy appearance.

Continue the topical remedies, and regulate her bowels with Sennæ Infus. p. r. n.

August 7th.—Quite well.

Case III.—Mary Last, ætat. seven, a delicate child, of a scrofulous habit, attacked with purulent ophthalmia of the left eye, of an acute character, for which bleeding, generally and locally, was used, together with tartar emetic to keep up nausea, but without checking the violence of the inflammation: sloughing of the cornea followed, and the sight was destroyed.

On the 30th of July, the right eye (which had some months previously been affected with strumous ophthalmia,) was attacked with the same violent disease, and threatened as speedy a course of mischief as the left eye had undergone. The child was now considerably reduced in strength by the remedies used to relieve the inflammation of the left eye. It was at this time that the opinion of Mr. Guthrie was requested. The lids were much swollen; the superior one was overlapping the inferior, accompanied with a scanty secretion of pus. On raising the superior lid, the cornea, having a clouded appearance, was seen diminished in circumference, from chemosis of the conjunctiva, which was raised in a rolled form around it. The child was pallid, with a weak wiry pulse, and expressed great pain when the eye was touched.

Mr. Guthrie advised twenty leeches to the neighbourhood of the eye, and the drain of blood from the orifices to be kept up as long as possible; and cold applications to the part.

31st of July.—The eye much the same. The leeches repeated, and a solution of the chloruret dropped into the eye three times a-day. The chemosis of the conjunctiva freely excised with the scissors. Mr. G. again saw the child, advised a continuance of the same plan, the application of twelve leeches, cupping on the temple to the amount of four ounces, and a purgative. The bleeding to be kept up during the night, and fresh leeches to be applied, if necessary to its continuance.

August 1st.—The eye better; a more abundant purulent discharge. The conjunctiva looks paler, and the chemosis is much lessened.

The Chloruret of Calcium and cold applications continued.

2d.—The eye improved. Mr. G. considered it safe.

The Chloruret continued, and four leeches applied.

The child was now extremely reduced in strength; but the pulse was less frequent and softer, the conjunctiva much paler, and the discharge of a plumy character, hanging in shreds to the surface of the cornea.

From the 3d of August, the eye continued to improve. The child was able to raise the superior palpebra, and two ulcerations were detected,—one extending along the inferior half of the cornea, and the other on the outer side of the superior half: they were perfectly clear, and free from any sloughy appearance. The chloruret was continued, and light nourishment administered.

August 10th.—The ulcerations look smaller, and seem to be healing, a slight haziness appearing round the edges. The child is able to open the eye, and the lids are free from granulations. The discharge is now very trifling, and the general health improving.

The child has had about fifty leeches applied, besides the loss of four ounces of blood by cupping.

